# *ylmD* and *ylmE* genes are dispensable for growth, cross-wall formation and sporulation in *Streptomyces venezuelae*

**DOI:** 10.1016/j.heliyon.2017.e00459

**Published:** 2017-11-21

**Authors:** Fernando Santos-Beneit, Jing-Ying Gu, Ulrich Stimming, Jeff Errington

**Affiliations:** a Centre for Bacterial Cell Biology, Medical School, Newcastle University, Newcastle Upon Tyne, UK; b School of Chemistry, Newcastle University, Newcastle Upon Tyne, UK

**Keywords:** Microbiology, Cell biology

## Abstract

Streptomycetes are Gram-positive filamentous soil bacteria that grow by tip extension and branching, forming a network of multinucleoid hyphae. These bacteria also have an elaborate process of morphological differentiation, which involves the formation of an aerial mycelium that eventually undergoes extensive septation into chains of uninucleoid cells that further metamorphose into spores. The tubulin-like FtsZ protein is essential for this septation process. Most of the conserved cell division genes (including *ftsZ*) have been inactivated in *Streptomyces* without the anticipated lethality, based on studies of many other bacteria. However, there are still some genes of the *Streptomyces* division and cell wall (*dcw*) cluster that remain uncharacterized, the most notable example being the two conserved genes immediately adjacent to *ftsZ* (i.e. *ylmDE*). Here, for the first time, we made a *ylmDE* mutant in *Streptomyces venezuelae* and analysed it using epifluorescence microscopy, scanning electron microscopy (SEM) and atomic force microscopy (AFM). The mutant showed no significant effects on growth, cross-wall formation and sporulation in comparison to the wild type strain, which suggests that the *ylmDE* genes do not have an essential role in the *Streptomyces* cell division cycle (at least under the conditions of this study).

## 1 Introduction

**Fig. 1:**
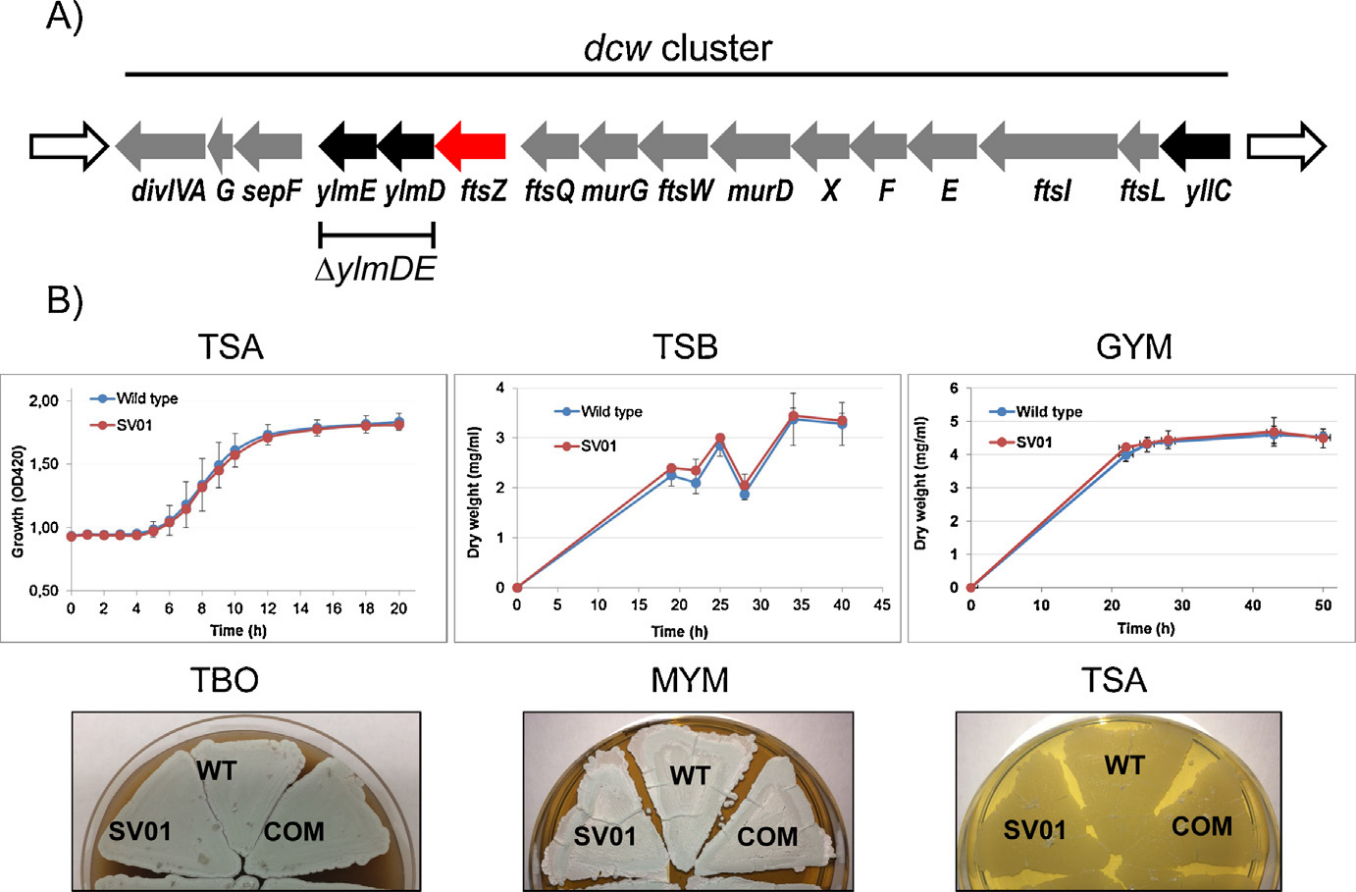
**A)** Map of the *Streptomyces venezuelae dcw* genomic region. Genes which have not been studied yet are shown in black (including the two genes of this study and excluding the *mur* genes). In order to highlight *ftsZ* we have shown the gene in red. The G refers to *sepG* and the X, F, E to *murX*, *murF* and *murE* genes. **B)** Phenotypic analyses of the strains constructed in this study. In all cases the cells on these plates were grown at 30 °C for a minimum of 4 days. Please, see Material and Method section for details about the growth determinations and culture conditions.

*Streptomyces venezuelae* is being promoted as a useful new model organism for study of the streptomycetes, which are the world’s most prolific producers of natural product molecules for the drug and agrochemical industries (Bibb et al., 2012; Bush et al., 2013). Production of natural products is often tightly connected to the developmental cycle, which includes the switch from the branching mycelial form to production of aerial hyphae and spores (McCormick and Flärdh, 2012). Improved understanding of how streptomycetes organise the major events of cell cycle progression is important for manipulating their growth under fermentative conditions in bioreactors, as well as controlling the production of secondary metabolites. Although several groups have been working on various aspects of the cell cycle in *Streptomyces*, much less is known about this general area of biology in comparison with bacteria such as *Escherichia coli* and *Bacillus subtilis* (Adams and Errington, 2009). However, *Streptomyces* biology brings with it a number of very interesting problems that are not addressable in those other organisms, including cell wall growth at the tips of filaments (rather than the more common intercalating MreB-dependent mode of cylindrical elongation) or the non-essentiality of FtsZ and many others cell division players (Flärdh, 2003; McCormick et al., 1994). FtsZ acts at an early stage of cell division by polymerizing into a ring at the site of septum formation and then serving as a scaffold for the assembly of the cell division apparatus (Adams and Errington, 2009). In most bacteria, the shut-down of *ftsZ* expression normally brings a lethal phenotype. However, *Streptomyces* constitutes an exception to this event. Both *Streptomyces coelicolor* and *S. venezuelae ftsZ*-null mutants are able to grow and to form aerial mycelium; although are unable to convert aerial hyphal filaments into spores (McCormick et al., 1994; Santos-Beneit et al., 2017). The *Streptomyces ftsZ* gene forms part of the well-conserved prokaryotic division and cell wall (*dcw*) gene cluster that includes genes required for efficient growth (*divIVA*), cell wall biosynthesis (*mur* genes) and cell division (*ftsZ, ftsQ*, *ftsW*, *ftsI*, *fstL*). In streptomycetes, as well as in other bacteria, most of the genes of the *dcw* cluster have been studied although, in some cases, very few details have been reported; like for example with the *mur* genes. In addition, some genes in this cluster remain completely unstudied, such as those immediately downstream of *ftsZ* (*ylmDE*) or the first gene of the cluster, *yllC* (see Fig. 1A). *ylmD* is particularly interesting because it overlaps with the *ftsZ* ORF, as it is the case in many other streptomycetes. Here we focus on the isolation of a *S. venezuelae* ATCC10712 Δ*ylmDE* double mutant and in the characterization of its mutant phenotype using epifluorescence microscopy, scanning electron microscopy (SEM) and atomic force microscopy (AFM).

## 2 Results

### 2.1 Construction and validation of a *S*. *venezuelae* Δ*ylmDE* mutant

Δ*ylmDE* (ΔSVEN_1736-35) was constructed by the ‘Redirect’ PCR targeting method of Gust et al. (2003). The two genes were first replaced with a selectable marker in the context of a cosmid containing a relevant segment of the *S. venezuelae* chromosome (the replaced segment extends from +5 of the *ylmD* ORF to the stop codon of *ylmE*; i.e., the 4 overlapping nucleotides of *ylmD* and *ftsZ* remain intact). The resulting disrupted cosmid was introduced into *S. venezuelae* by conjugation with the expectation that null mutant derivatives would be generated by double crossing over. Mutants with the correct genotype were identified by their apramycin-resistant and kanamycin-sensitive phenotypes (Kan^S^/Apra^R^) during two rounds of selection (see Material and Methods). Four of these recombinants were selected for further PCR analyses using different combinations of primers, either hybridizing within SVEN_1735 (*ylmE*) and SVEN_1736 (*ylmD*) or within the apramycin resistance *aac*(3)IV cassette, as well as hybridizing upstream and downstream of these genes (see Supplementary Fig. S1). A representative isolate with the correct structure was designated SV01.

We began to examine this mutant by checking its growth in different media (Fig. 1B). No phenotypic differences were observed between the parental and mutant strains in TSB (Tryptone Soya Broth, Oxoid), TSA (Tryptone Soya Agar, Oxoid), GYM (glucose, yeast extract and malt extract), MYM (maltose, yeast extract, malt extract and agar) or TBO medium (tomato puree, oat meal and agar).

### 2.2 *ylmDE* is not required for cross-wall formation

**Fig. 2:**
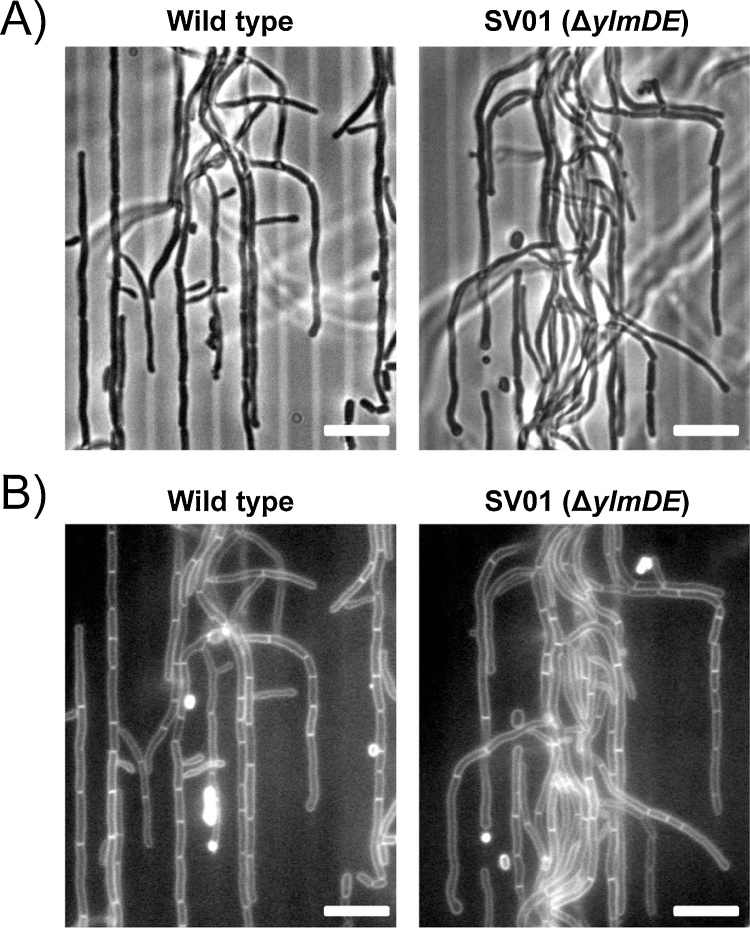
Cross-wall analyses in wild type and Δ*ylmDE* strains. **A)** Panels show brightfield images. **(B)** Panels show FM4-64 stained membrane images. The cells were grown in a home-made microfluidic device with GYM medium (supplemented with 0.5 μg. ml^−1^ of FM4–64) during a similar period of time (i.e. 18 h). Scale bars = 5 μm.

Streptomycetes grow vegetatively as branching hyphal filaments with only occasional cross-walls, but undergo multiple septation when these bacteria start morphological differentiation (Schlimpert et al., 2016). We examined whether the mutant was able to make cross-walls in vegetative cells. To this aim we grew wild type and mutant strains using a microfluidic device. The medium was supplemented with FM4-64 dye, which allows the visualization of cross-walls by staining the membrane layer of these structures. As shown in Fig. 2, the mutant strain was able to make cross-walls in a similar manner as the wild type.

### 2.3 Δ*ylmDE* sporulates to a similar extent than the wild type although makes more heterogeneous and longer spores

**Fig. 3:**
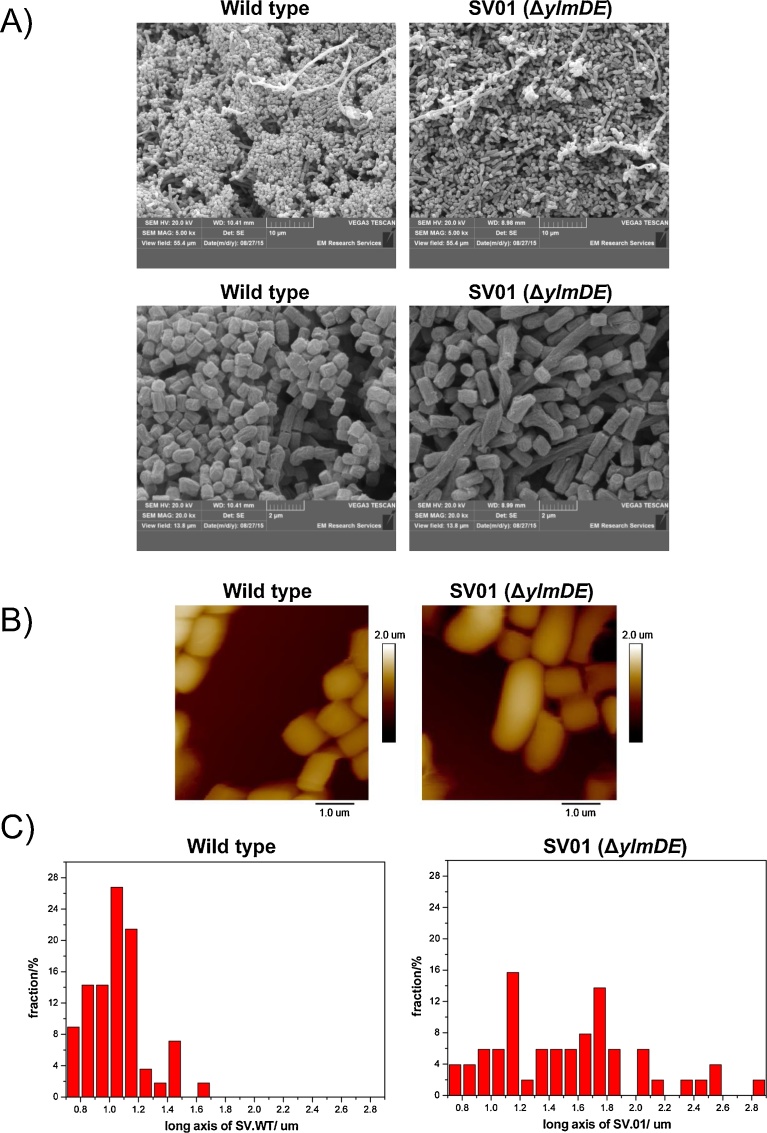
Sporulation analyses of wild type and Δ*ylmDE* strains. **A)** Scanning electron microscopy images of wild-type and SV01 (Δ*ylmDE*) mutant cells grown in TBO medium during 6 days. Magnifications of 5000 (above) and 20000 (below) are shown for each strain. **(B)** Typical AFM images of wild type and Δ*ylmDE* spores. Cell-Tak allows the spores to stick firmly to the HOPG surface**. (C)** Histograms of the long axis of wild type and Δ*ylmDE* spores. Spores were analysed from a total area of 560 μm2 (from 14 samples) with more than 50 spores being evaluated for each strain.

Next we examined whether the mutant was affected in sporulation, as it is the case for mutants of several genes in the *dcw* cluster. To this aim the strains were grown on TBO, a solid medium in which *S. venezuelae* sporulates to near-completion (Higgens et al., 1974). Visually, the sporulation efficiency was indistinguishable for Δ*ylmDE* and wild type strains, including the production of the typical pigment associated with the sporulation process (Fig. 1B). Samples taken directly from the TBO cultures were imaged by scanning electronic microscopy. Although the morphology of the spores appeared slightly more heterogeneous in Δ*ylmDE* than in the wild type strain, the experiment clearly showed that the mutant was able to sporulate in a similar extent than the parental strain (see Fig. 3A). To quantify the size of the wild type and mutant spores we used AFM in fluid, which allows bio-imaging the cells in physiological condition and avoids the dehydration associated with SEM (see Fig. 3B). The length of the short axis of the spores was similar between the two strains (0.6–1.0 μm). However, the long axis was more variable in the mutant than in the wild type (0.7–2.9 vs 0.7–1.7 μm, respectively) as well as being, on average, longer for spores of the Δ*ylmDE* mutant (WT = 1.0 μm ± 0.20; SV01 = 1.6 μm ± 0.62; see Fig. 3C). Genetic complementation did not restore the observed phenotype (COM = 1.8 μm ± 0.49), therefore the SV01 phenotype might be associated with a transcriptional polarity effect.

## 3 Discussion

We analysed well conserved *dcw* genes by isolating a *ylmDE* double mutant for *Streptomyces*. These two genes form part of the prokaryotic *dcw* gene cluster that includes *ftsZ* and other key players required for efficient growth, cell wall biosynthesis, cell division and sporulation. Most of the conserved cell division genes of this cluster have been inactivated in *Streptomyces* without the anticipated lethality, based on studies of many other bacteria. Actually, the full essentiality of *ftsZ* reported in most bacteria is not observed in *Streptomyces* (McCormick et al., 1994; Santos-Beneit et al., 2017). Despite the importance of this cluster, there are still some genes of the *Streptomyces dcw* cluster that remain unstudied, the most notable example are the two conserved genes (*ylmDE*) immediately adjacent to *ftsZ*. SVEN_1735 (*ylmE*) and SVEN_1736 (*ylmD*) have conserved domains for Type III pyridoxal 5-phosphate (PLP)-dependent enzymes (cl00261) and multi-copper polyphenol oxidoreductase laccases (pfam02578), respectively. Therefore, it is difficult to relate the function of these genes with the cell cycle process just by their protein product sequence conservation.

In this study we have shown that a Δ*ylmDE* mutant is able to sporulate and grow in a similar manner to the wild type, which indicates that these genes, in spite of being clustered together with *ftsZ*, are not essential for the normal development of the bacterium. The result is in agreement with previous studies with *Synechocystis* PCC6803 and *Streptococcus pneumoniae* in which the *ylmDE* genes were shown to be dispensable to cell growth and division (Marbouty et al., 2009; Fadda et al., 2003). In *S. pneumoniae* inactivation of *ylmE* (*ylmD* is missing from this organism) produced cells similar in shape to the wild type but, on average, the mutant cells were slightly larger than wild-type cells (Fadda et al., 2003). In this study, using high resolution microscopy techniques (SEM and AFM) we have noted that the Δ*ylmDE* mutant makes more heterogeneous and longer spores than the wild type, which points to a possible role of these genes in control of the correct placement of the sporulative septa. However, the complementation vector was not able to restore the Δ*ylmDE* mutant phenotype which suggests a possible transcriptional polarity effect of the deletion cassette over the downstream genes (*sepF*, *sepG* and *divIVA*) rather than a role of the proteins in the control of the sporulative septa placement. Nevertheless, it is unknown if the enzymes encoded by *ylmD* and/or *ylmE* could play a role in the cell division process under other testing conditions, perhaps, for its ability to respond to changing nutritional signals and transmit this information directly to the cell-cycle machinery. Further analyses will be required to characterize the specific role of *ylmDE* in the cell but, in summary, whereas *ftsZ* is essential in streptomycetes for cross-wall formation and sporulation, deletion of its two conserved downstream genes (*ylmDE*) caused no gross changes to growth and sporulation in *S. venezuelae*.

## 4 Materials and methods

### 4.1 Bacterial strains, constructs and growth conditions

All the strains, plasmids and primers used in this work are listed in Table 1. The wild type *S. venezuelae* strain was ATCC10712. The ‘Redirect’ PCR targeting method was used for the construction of the Δ*ylmDE* mutant (Gust et al., 2003). SVEN_ 1735–36 genes have a central localization on cosmid SV-4-G-01 which is positive for the double-crossover event, therefore this cosmid was selected for this study. A PCR with primers FSB03 and FSB04 was used to generate the recombination substrate (including the apramycin resistance cassette and homologous flanking regions to the SVEN_ 1735–36 genes) for replacement of *ylmDE* in cosmid SV-4-G-01. The PCR product was transformed into *E. coli* BW25113/pIJ790/SV-4-G-01 as described previously (Gust et al., 2003). Apramycin-resistant transformants were selected, and the recombinant cosmid was identified by PCR and sequencing. Then, the cosmid was introduced into *S. venezuelae* via *E. coli* ET12567 (pUZ8002) mediated conjugation. Selected exconjugants were screened for Kan^S^/Apra^R^ phenotypes and checked by PCR. For performing the complementation analyses it was first constructed an integrative vector carrying the *S. venezuelae ftsZ* promoter with an introduced NdeI-EcoRV cloning site (i.e. pFtsZp-neo). The *ftsZ* promoter region was amplified by PCR using total DNA as template. The primers FSB09 and FSB10 amplified a 306 bp fragment encompassing the entire promoter region of the gene (Flärdh et al., 2000). The BamHI (FSB09) and NdeI (FSB10) cloning sites were introduced in the primer sequences. Then, a BamHI-NdeI fragment was cloned into pLUXAR-neo (Santos-Beneit et al., 2009) obtaining pFtsZp-neo. *ylmDE* fragment was amplified by PCR using the primers FSB41 and FSB42. The NdeI recognition sequence was introduced in the forward primer and the EcoRV in the reverse primer. The NdeI-EcoRV digested PCR product was cloned into pFtsZp-neo giving pFtsZp-*ylmDE*. The cloned insert was confirmed by sequencing.

**Table 1:** Strains, plasmids and primers used in this work.

Bacterial strains	Description	Reference
*E. coli* DH5α	F′Ф80 dLacZ ΔM15	Hanahan, 1983
*E. coli* BW25113 (pIJ790)	K-12 derivative (Δ*araBAD*, Δ*rhaBAD*) carrying plasmid pIJ790	Datsenko and Wanner, 2000
*E. coli* ET12567 (pUZ8002)	*dam, dcm, hsdS, cat, tet*, carrying helper plasmid pUZ8002	MacNeil et al., 1992
*S. venezuelae* ATCC10712	Parental strain	ATCC
*S. venezuelae* SV01	*S. venezuelae* Δ*ylmDE*:aac(3)IV	This work
*S. venezuelae* COM	*S. venezuelae* Δ*ylmDE* + pFtsZp-*ylmDE*	This work
**Plasmids or Cosmids**	**Description**	**Reference**
pIJ773	Vector containing aac(3)IV-oriT	Gust et al., 2003
Sv-4-G01/Δ*ylmDE*:aac(3)IV-oriT	*S. venezuelae* cosmid deleted in *ylmDE*	This work
pLUXAR-neo	Integrative vector-phiC31, *aac(3)IV, neo*	Santos-Beneit et al., 2009
pFtsZp-neo	*ftsZ* promoter into pLUXAR-neo	This work
pFtsZ-*ylmDE*	*ylmDE coupled to ftsZp* into pFtsZp-neo	This work
**Primers**		
**FSB03:** CCAGGCCGAAGAGCTGGACGTCCCGGACTTCCTGAAGTGATTCCGGGGATCCGTCGACC (Redirect)		
**FSB04:** CATATTTTCTGCTGTGGTCCGACTTGCTTCGCGACGTTATGTAGGCTGGAGCTGCTTC (Redirect)		
**FSB05:** CCGTGCACCTGGTTCATCCAGAC (rev inside SVEN_1736 ORF)		
**FSB07:** GCCGAGGTAGACCGCCATCTTG (rev inside SVEN_1734)		
**FSB09:** CCGCTGGATCCGGGAGTTGACGGGTATCAC (dir for *ftsZ*p, BamHI)		
**FSB10:** GCTGCCATATGGAAGGCCTCTCGCCTCGAGTTAC (rev for *ftsZ*p, NdeI)		
**FSB16:** GCCTGTGCCGACCTTGATCTGAC (dir inside SVEN_1735 ORF)		
**FSB19:** GACCTCGGTCTCTTCGAGATCAAC (dir inside *ftsZ*)		
**FSB21:** GCGAGTGAGGTGGCAGGGGCAATG (rev inside aac(3)IV ORF)		
**FSB22:** GGTGTGCTGCTGGTCCACAGCTC (dir inside aac(3)IV ORF)		
**FSB41:** CTTCCGCATATGATAGGACAGCGCTTCGACGCGAAC (dir for SVEN_1736-35, NdeI)		
**FSB42:** GCCCGATATCCACGGGTCATGCCGCCGTTCTG (rev for SVEN_1736-35, EcoRV)		

*S. venezuelae* strains were grown on TBO medium (tomato puree, oat meal and agar) to make spore glycerol stocks as well as for the SEM and AFM analyses. For growing the cells in liquid or solid conditions TSB (Tryptone Soya Broth, Oxoid), TSA (Tryptone Soya Agar, Oxoid), GYM (glucose, yeast extract and malt extract) and MYM (maltose, yeast extract, malt extract and agar) were used. Quantification of growth in the *S. venezuelae* solid TSA cultures was performed using black polystyrene sterile 96-well plates. In each well (containing 100 μL of medium) was added 4 μL of a stock dilution containing 10^6^ spores. Plates were then incubated at 30 °C during 20 h. The growth was determined by optical density (420 nm) using a BMG Fluostar Optima fluorometer. *S. venezuelae* liquid cultures were performed in 100 ml of medium using 500 ml baffled flasks. The cultures were inoculated with 10^6^ spores per ml and incubated at 30 °C in an orbital shaker for reproducible and dispersed growth during 40–50 h. Samples for growth determination from the liquid TSB cultures were taken after 19, 22, 25, 28, 34 and 40 h of incubation. Samples for growth determination from the liquid GYM cultures were taken after 22, 25, 28, 43 and 50 h of incubation. Growth was determined by dry weight (culture samples of 2 ml were washed twice with MilliQ water and dried during 4 days at 80 °C before the weight measurements).

### 4.2 Fluorescence microscopy

To visualise growing mycelia and cross-wall formation in wild type and Δ*ylmDE* strains, GYM cultures were supplemented with 0.5 μg. ml^-1^ of FM4–64 (Molecular Probes). The cells were grown in an agarose-pad based microfluidic device as described in Eland and co-workers (2016). Images were acquired using a Nikon Ti microscope equipped with a Nikon Plan Apo x100/1.4 oil objective and FRAP-AI v.7.7.5.0 software (MAG Biosystems, Molecular Devices) and analysed using the ImageJ software (NIH).

### 4.3 Scanning electron microscopy (SEM)

Scanning electron microscopy was performed using a VEGA3 (TESCAN) Scanning Electron Microscope. Before the SEM analysis, glutaraldehyde-fixed samples were washed in phosphate buffered saline, dehydrated through a graded ethanol series and critical-point dried with carbon dioxide. Finally, samples were mounted on aluminium stubs and coated with a gold sputter coater.

### 4.4 Atomic force microscopy (AFM)

AFM measurements were performed in 10 mM PBS buffer solutions (pH = 7) using a Bruker Multimode 8 Microscope in Scanasyst mode with Bruker Scanasyst-Fluid+ tip. Cell-Tak (SLS Life Science) was used as adhesive protein to facilitate a firm contact between bacterial spores and HOPG surface (Bruker, ZYB quality) for AFM imaging. These adhesive proteins were applied to the HOPG surface by mixing 1 μl Cell-Tak with 0.5 μl 1 M sodium hydroxide and 28.5 μl 0.1 M sodium bicarbonate. The solution was immediately added to a freshly cleaved HOPG surface, and left for 20 mins at room temperature. The surface was then rinsing with deionized water (Elga Option-Q water purifier, resistivity > 18.2 MΩ, total organic carbon content < 10 ppb) to remove excess Cell-Tak solution. Bacterial spores were immobilized by adding 30 μl spore suspension to Cell-Tak coated HOPG surface, incubating for at least 2 hours, and removing non-adhering spores by gently rinsing the surface with deionized water. To make the histograms of the axis of the spores more than 50 spores of each strain were evaluated.

## Declarations

### Author contribution statement

Fernando Santos-Beneit, Jing-Ying Gu, Ulrich Stimming, Jeff Errington: Conceived and designed the experiments; Performed the experiments; Analyzed and interpreted the data; Contributed reagents, materials, analysis tools or data; Wrote the paper.

### Funding statement

This work was supported by grant 098374 from the Wellcome Trust to JE.

### Competing interest statement

The authors declare no conflict of interest.

### Additional information

Supplementary content related to this article has been published online at http://dx.doi.org/10.1016/j.heliyon.2017.e00459.

No additional information is available for this paper.

## Appendix A Supplementary data

Supplementary Figure S1 PCR analyses of the Δ*ylmDE* mutant strains. **A)** The expected amplification products sizes were obtaining in the wild type strain with the next pair primers: FSB19 (forward primer that hybridizes upstream of the SVEN_1736 ORF) / FSB05 (reverse primer that hybridizes within the SVEN_1736 ORF) and FSB16 (forward primer that hybridizes within the SVEN_1735 ORF) / FSB07 (reverse primer that hybridizes downstream of the SVEN_1735 ORF). These products were not observed in any of the Δ*ylmDE* selected mutants (SV01, SV02, SV03 and SV04). On the contrary, the correct amplification products sizes were obtaining in the Δ*ylmDE* selected mutants with the next pair primers: FSB19 (forward primer that hybridizes upstream of the SVEN_1736 ORF) / FSB21 (reverse primer that hybridizes within the *aac*(3)IV ORF) and FSB22 (forward primer that hybridizes within the the aac(3)IV ORF) / FSB07 (reverse primer that hybridizes downstream of the SVEN_1735 ORF). These products were not obtained in the wild type strain. For simplicity the gel electrophoresis analyses are not shown in the Figure. **B)** The Δ*ylmDE* selected mutants were also checked by PCR using flanking forward and reverse primers to SVEN_1736-35 (i.e. primers FSB19 and FSB07). Because the wild type and Δ*ylmDE* respective amplifications products were similar in size with these primers (2051 bp and 1956 bp, respectively) the PCR products were further digested with *MluI* (which cuts within *aac*(3)IV but not within the SVEN_1736-35 genes); giving two bands in the mutant strain (i.e. 1465 bp and 491 bp) and one band in the wild type (i.e. 2051 bp). The gel electrophoresis analysis showing the run of these *MluI* digested PCR products is shown at the right of the panel.
