# Supplementary figures and images for: ylmD and ylmE genes are dispensable for growth, cross-wall formation and sporulation in Streptomyces venezuelae
Source: Heliyon. 2017 Nov 21;3(11):e00459. doi: 10.1016/j.heliyon.2017.e00459 (PMC5701809; doi:10.1016/j.heliyon.2017.e00459)

## Slide 1
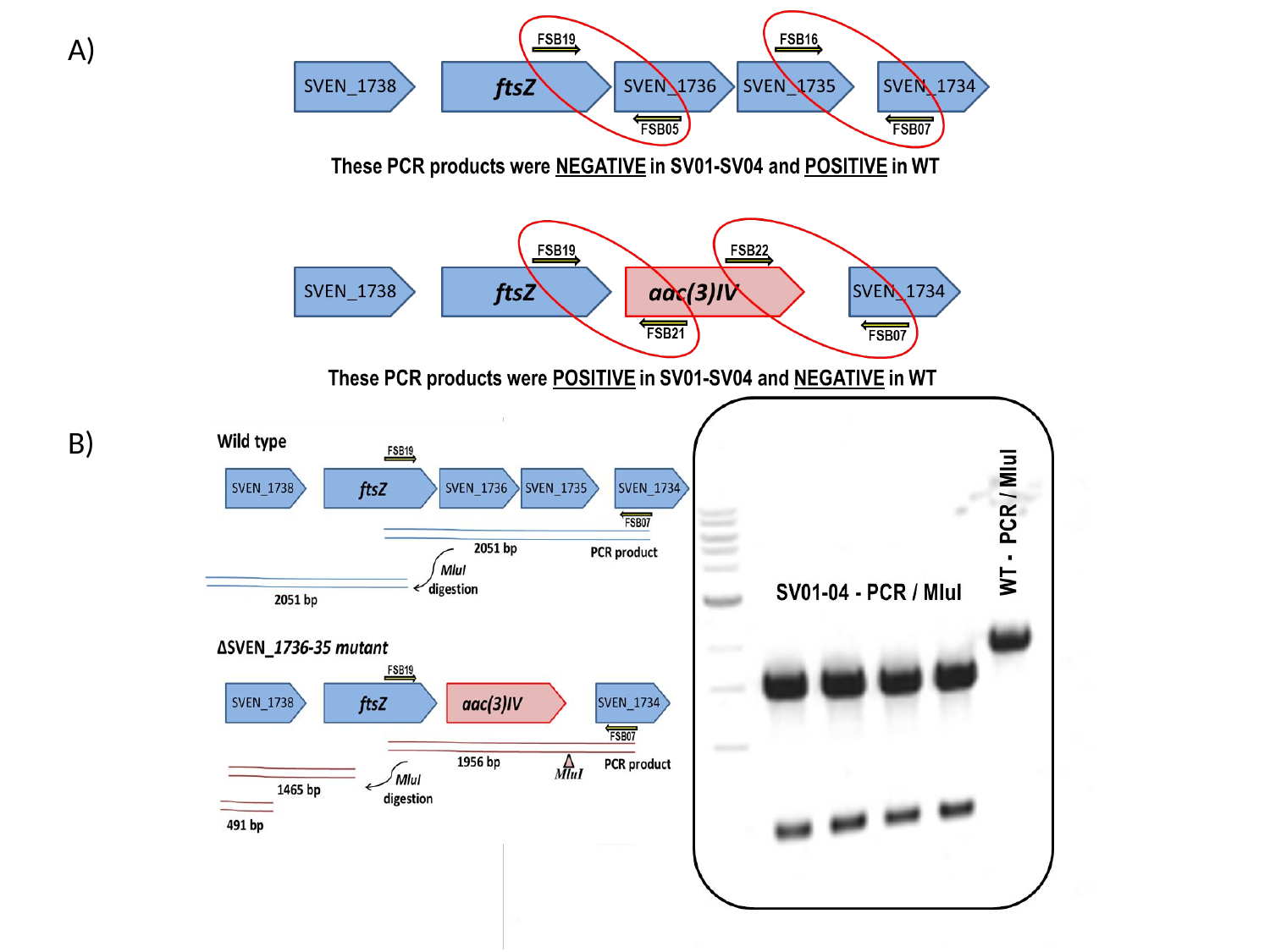

A)
B)

Supplement: Supplementary Figure S1 — PCR analyses of the ΔylmDE mutant strains. A) The expected amplification products sizes were obtaining in the wild type strain with the next pair primers: FSB19 (forward primer that hybridizes upstream of the SVEN_1736 ORF) / FSB05 (reverse primer that hybridizes within the SVEN_1736 ORF) and FSB16 (forward primer that hybridizes within the SVEN_1735 ORF) / FSB07 (reverse primer that hybridizes downstream of the SVEN_1735 ORF). These products were not observed in any of the ΔylmDE selected mutants (SV01, SV02, SV03 and SV04). On the contrary, the correct amplification products sizes were obtaining in the ΔylmDE selected mutants with the next pair primers: FSB19 (forward primer that hybridizes upstream of the SVEN_1736 ORF) / FSB21 (reverse primer that hybridizes within the aac(3)IV ORF) and FSB22 (forward primer that hybridizes within the the aac(3)IV ORF) / FSB07 (reverse primer that hybridizes downstream of the SVEN_1735 ORF). These products were not obtained in the wild type strain. For simplicity the gel electrophoresis analyses are not shown in the Figure. B) The ΔylmDE selected mutants were also checked by PCR using flanking forward and reverse primers to SVEN_1736-35 (i.e. primers FSB19 and FSB07). Because the wild type and ΔylmDE respective amplifications products were similar in size with these primers (2051 bp and 1956 bp, respectively) the PCR products were further digested with MluI (which cuts within aac(3)IV but not within the SVEN_1736-35 genes); giving two bands in the mutant strain (i.e. 1465 bp and 491 bp) and one band in the wild type (i.e. 2051 bp). The gel electrophoresis analysis showing the run of these MluI digested PCR products is shown at the right of the panel. [file mmc1.pptx]
